# Diverticulitis and Diverticulosis of the Appendix: A Case Series

**DOI:** 10.7759/cureus.30786

**Published:** 2022-10-28

**Authors:** Omotara Kafayat Lesi, Spencer Probert, Muhammad Rafaih Iqbal, Obiora Mann Ajuluchukwu, Mojolaoluwa Olugbemi, Noreen Rasheed, Bryony Lovett, Philip Idaewor, Dennis Wayne Chicken, Abdalla Saad Abdalla Al-Zawi

**Affiliations:** 1 General and Colorectal Surgery, Basildon University Hospital, Essex, GBR; 2 General Surgery, Basildon & Thurrock University Hospital, Basildon, GBR; 3 Colorectal Surgery, Basildon and Thurrock University Hospitals, Mid and South Essex NHS Foundation Trust, Essex, GBR; 4 Radiology, St George's Hospital, London, GBR; 5 Colorectal Surgery, West Suffolk Hospital, Suffolk, GBR; 6 Radiology, Basildon and Thurrock University Hospital, Essex, GBR; 7 General Surgery, Mid and South Essex NHS Foundation Trust, Basildon, GBR; 8 Histopathology/Cellular Pathology, Mid and South Essex NHS Foundation Trust, Basildon, GBR; 9 Histopathology/Cellular Pathology, Basildon and Thurrock University Hospital, Basildon, GBR; 10 General and Breast Surgery, Mid and South Essex University Hospital Group, Basildon, GBR; 11 General and Breast Surgery, Basildon and Thurrock University Hospital, Basildon, GBR; 12 General and Breast Surgery, Anglia Ruskin University, Chelmsford, GBR

**Keywords:** laparoscopic appendicectomy, computed tomography, diverticulosis, appendicectomy, acute appendicitis, appendiceal diverticulitis

## Abstract

Introduction

Diverticula of the appendix is a rare entity, may be complicated by inflammation/infection, and clinically mimics acute appendicitis. The reported associated risk factors include male gender, Hirschprung’s disease, cystic fibrosis and adult age, where some reports claim that they are also associated with an increased risk of appendiceal malignancy. Imaging has a place in pre-operative diagnosis, however, most of the cases were diagnosed during a pathological examination after surgery. They are associated with a higher rate of perforation (more than four times compared with classical acute appendicitis). In this review, we present a case series of five patients diagnosed with diverticulitis and one with diverticulosis of the appendix that were managed at a single centre. Our aim is to explore the common clinical, radiological, and intra-operative findings associated with this disease as well as the outcome of management.

Materials and methods

A total number of six cases of diverticular disease of the appendix diagnosed and managed at Basildon University hospital in the period between 2016 and 2020 were studied. The demographic details and clinical data including presenting symptoms, laboratory results, radiological characteristics, intraoperative findings and histopathological features were analysed.

Results

The study group included four males and two females, with an age range of 20-84 years. The most common presenting clinical symptoms were right iliac fossa abdominal pain, nausea, anorexia, and diarrhoea. Half of the cases showed a thickened appendix in the pre-operative CT scan. An inflamed or perforated appendix was seen in five cases as well as inflammation of the diverticula.

Conclusion

Appendiceal diverticulitis is an uncommon pathology that imitates acute appendicitis, and appendicectomy is the standard treatment. Prophylactic appendicectomy is recommended for non-inflamed diverticula - this is due to the potential risk of inflammation, perforation, and the risk of developing an appendiceal neoplasm.

## Introduction

Diverticulosis of the appendix is an uncommon pathology, first described by Kelynack in 1893 [1]. Its incidence is reported to be between 0.004% and 2.1% [2]. Symptoms of diverticulitis of the appendix are similar to those of acute appendicitis, which is the most common pathology of the appendix. Diverticulitis of the appendix is four times more likely to lead to perforation when compared to appendicitis and may be a sign of an underlying neoplasm [3]. Therefore, it is extremely important to distinguish diverticulitis of the appendix from appendicitis.

## Materials and methods

We present six cases of diverticular disease of the appendix diagnosed and managed in our unit, those cases were diagnosed in the period between 2016 and 2020. The clinical records for those cases were retrieved and demographic data, such as age and gender, was collected. Also, the clinical information related to the presenting features, such as abdominal pain, nausea, anorexia, and diarrhoea, was analyzed. The diagnostic work-up included inflammatory markers assessment and imaging studies. The intra-operative findings were also been reviewed in every case.

## Results

Case 1

**Figure 1:**
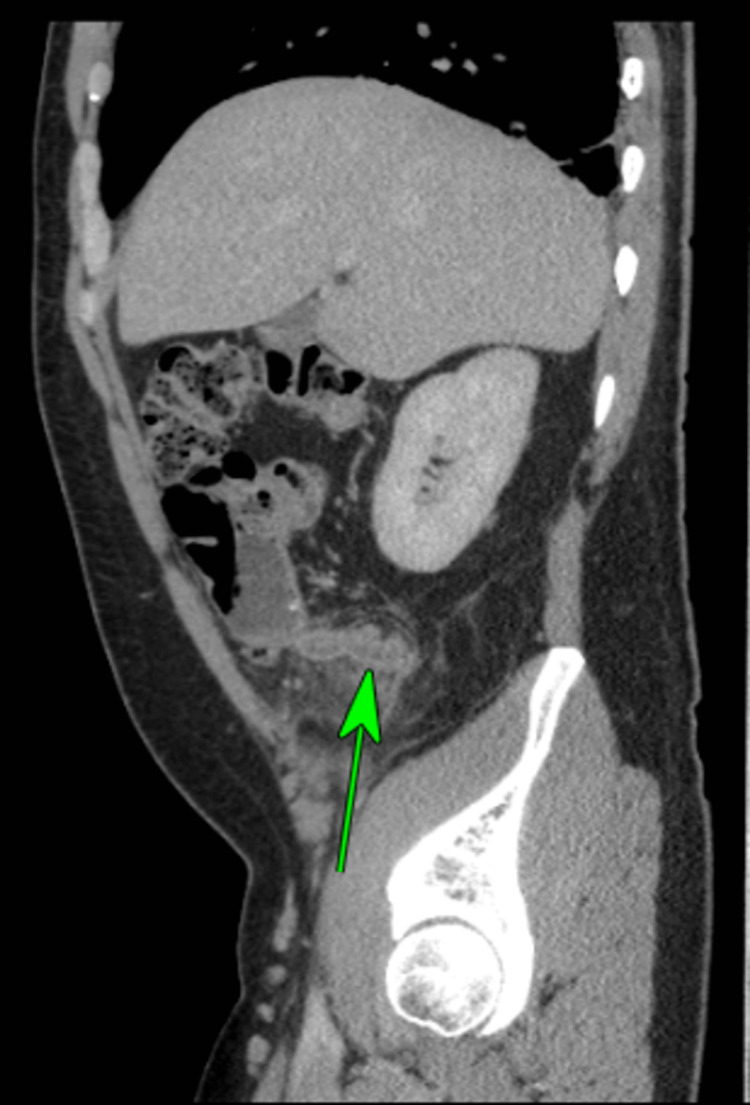
Sagittal CT Scan image of thickened appendix with extensive perifocal fat stranding.

**Figure 2:**
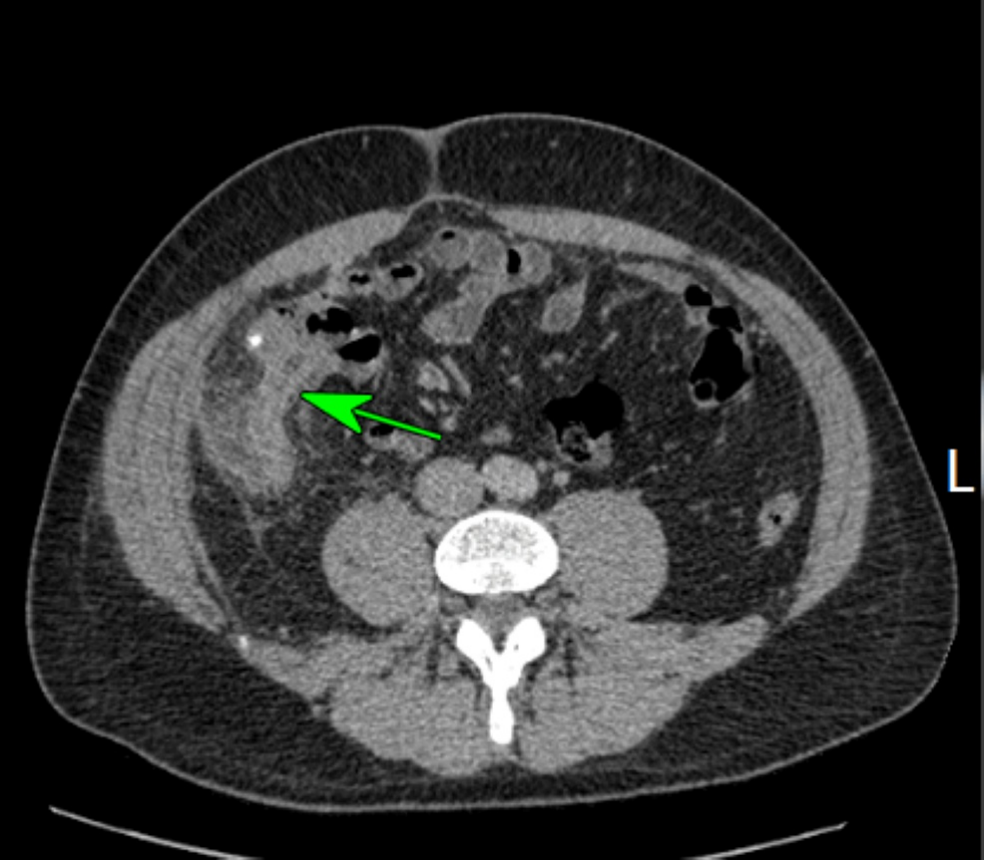
Axial image of thickened appendix with extensive perifocal fat stranding. A small calcific focus (possibly a displaced appendicolith) was visualised within the lumen of the caecum.

**Figure 3:**
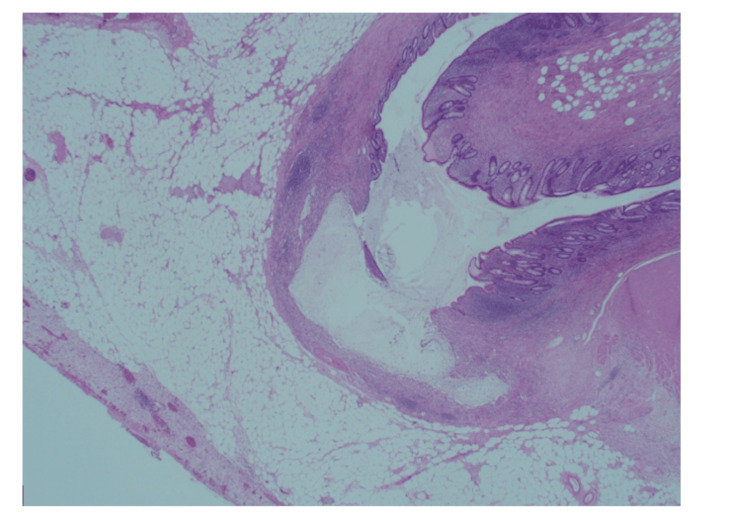
Appendicular diverticulum with associated mild active chronic inflammation.

**Figure 4:**
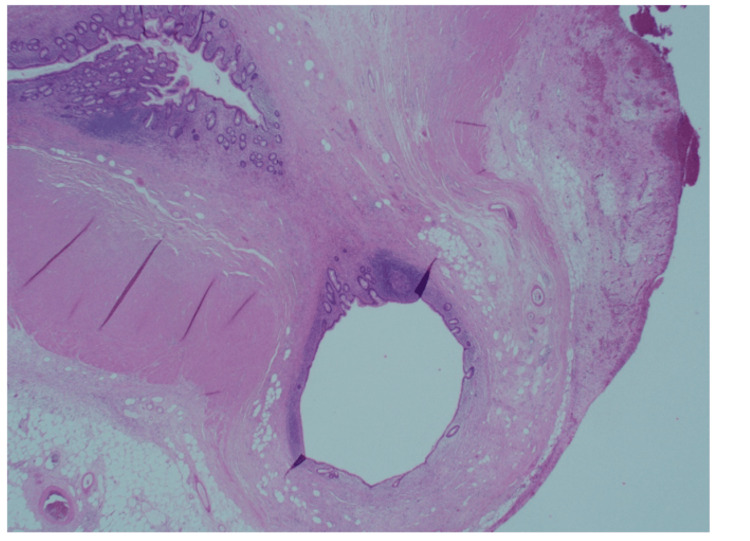
Part of the diverticula protrusion into the fibromuscular wall.

**Figure 5:**
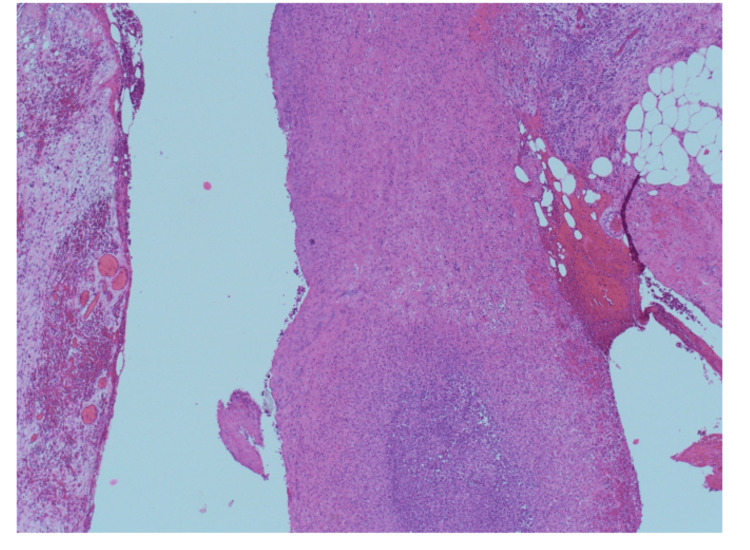
An area with severe active chronic inflammation with associated serositis.

Patient 1 was a 40-year-old male who presented with a two-day history of periumbilical abdominal pain, migrating to the right iliac fossa (RIF), nausea, and diarrhoea. On clinical examination, there was tenderness in the RIF with guarding. Initial blood investigations showed a C-reactive protein (CRP) of 288 mg/L and a white cell count (WBC) of 14x10^9^/L. Computed tomography (CT) reported a thickened appendix (diameter of 1.3 cm) with extensive perifocal fat stranding (Figures 1, 2). A small calcific focus (possibly a displaced appendicolith) was visualised within the lumen of the caecum. A few small lymph nodes were noted in the region. The patient subsequently underwent a laparoscopic appendicectomy. Intraoperatively, an inflamed retrocaecal appendix was noted, with adherent terminal ileum and a right para-colic abscess. Histological analysis of the appendix showed transmural acute inflammation and serositis with multiple diverticula. There was no evidence of parasites, dysplasia, or malignancy. The histological appearance was indicative of acute diverticulitis of the appendix (Figures 3, 4, 5).

Case 2

**Figure 6:**
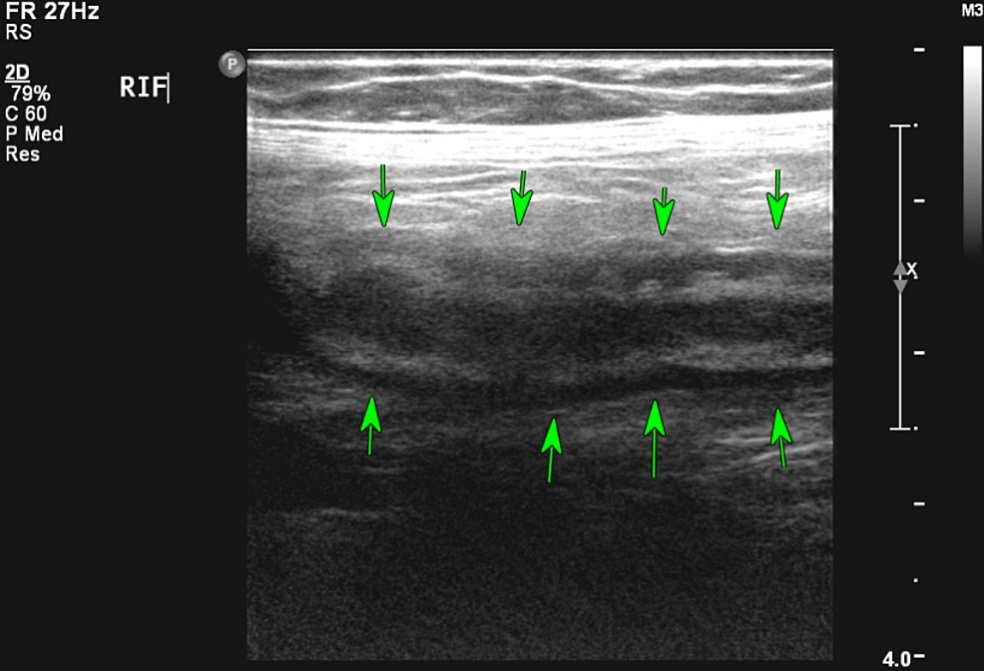
Thickened and oedematous, non-compressible blind-ended bowel loop in the right iliac fossa.

**Figure 7:**
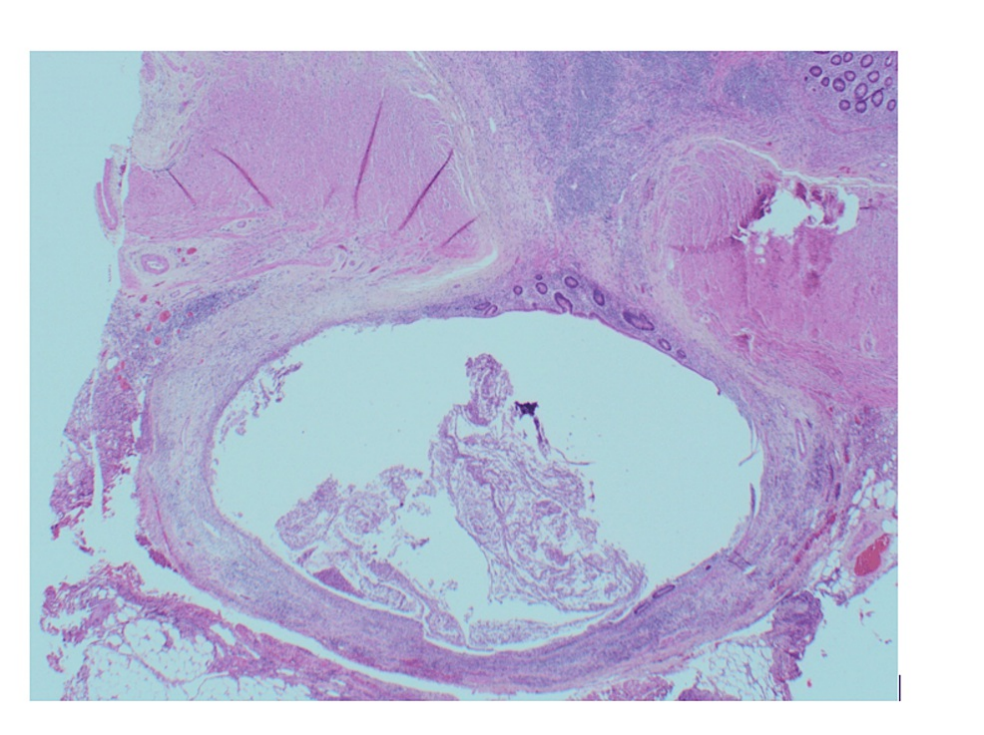
Diverticulum with associated fibrosis of wall and mild to moderate active chronic inflammation (x2).

Patient 2 was a 20-year-old female that presented with a one-day history of RIF pain and nausea. Clinically there was RIF tenderness. Initial blood investigations showed a CRP of 14 mg/L and a WBC of 10.4x10^9^/L. Ultrasonography revealed a thickened and oedematous, non-compressible blind-ended bowel loop in the RIF measuring 8 mm in diameter (Figure 6). The patient underwent a laparoscopic appendicectomy during which an inflamed appendix was found along with inflammatory fluid in the pelvis. Histologically the appendix showed mucosal inflammation not involving the muscle layer and an inflamed diverticulum (Figure 7).

Case 3

**Figure 8:**
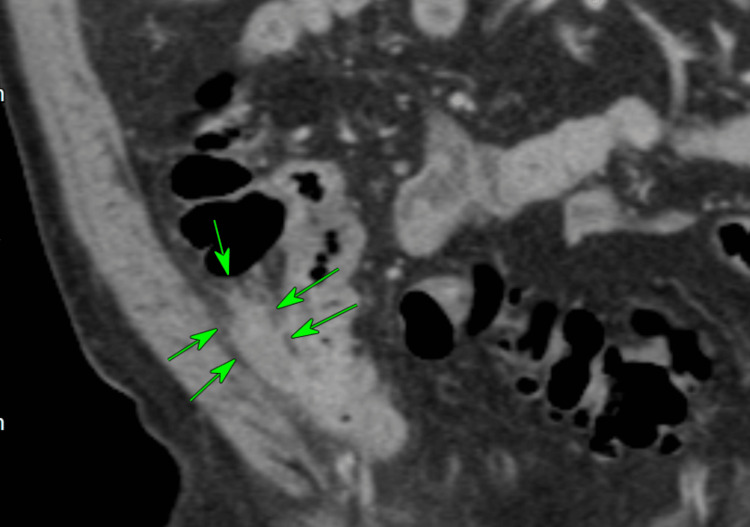
CT Scan coronal view showing thickened appendix associated with peri-appendiceal inflammatory changes within the RIF, consistent with acute appendicitis. RIF: right iliac fossa

**Figure 9:**
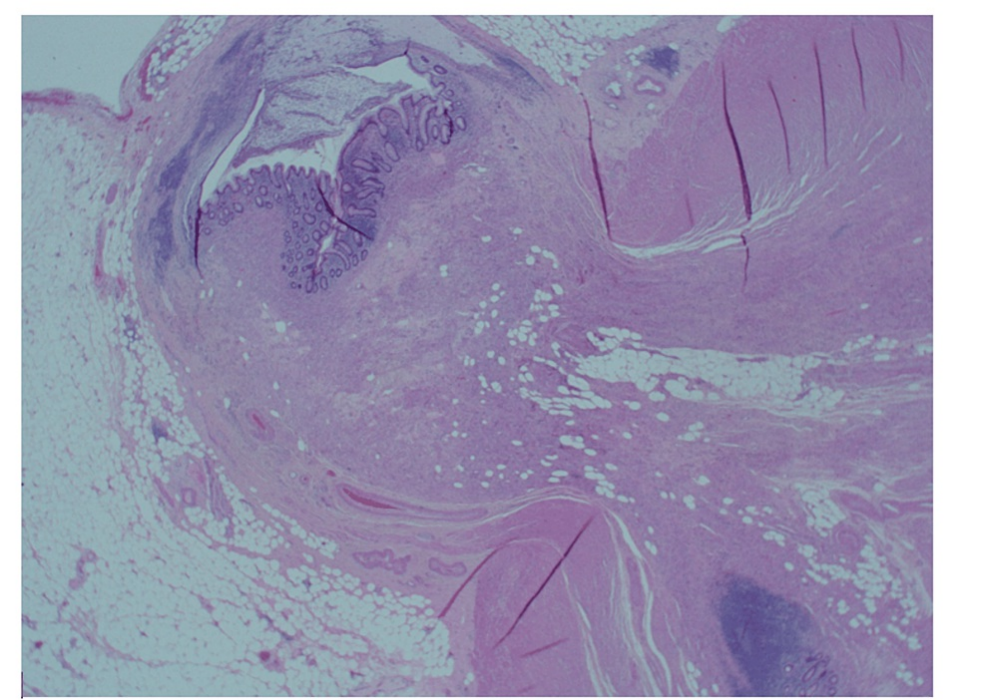
Deeply sited diverticula with associated mild acute inflammation.

**Figure 10:**
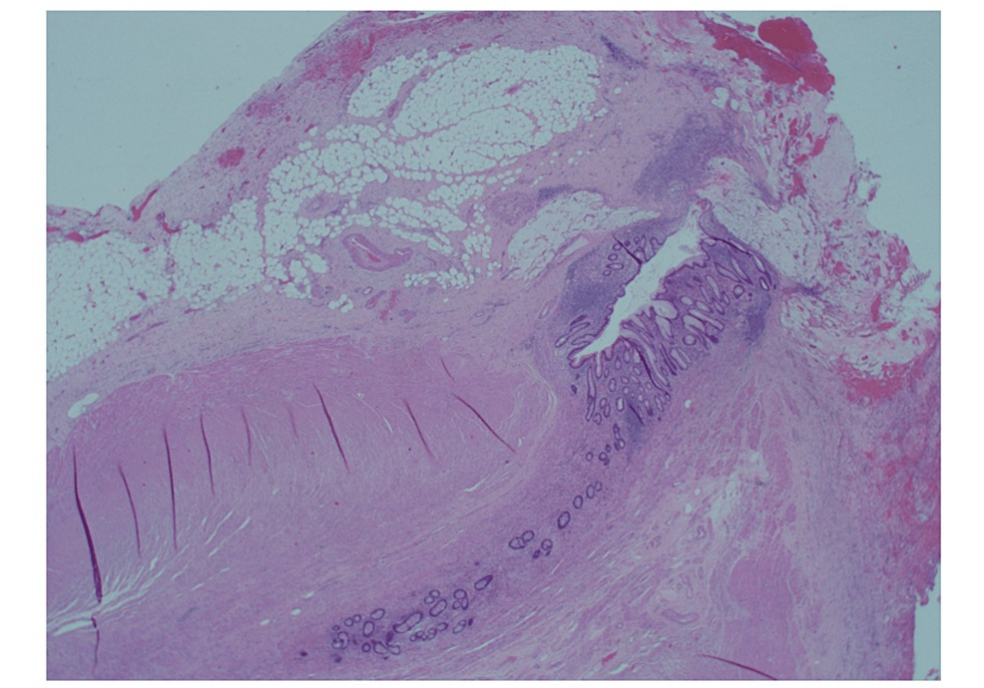
Diverticulum extends into the surrounding fat.

Patient 3, a 70-year-old male, presented with a three-day history of RIF pain. Clinically the patient had tenderness in the RIF and anorexia. Initial blood investigations showed a CRP of 19 mg/L, and a WBC of 10.7x10^9^/L. A CT scan noted a thickened appendix (diameter of 1 cm) associated with peri-appendiceal inflammatory changes within the RIF, consistent with acute appendicitis (Figure 8). Also noted was an adherent loop of the small bowel, with associated mild wall thickening likely to be reactive to the adjacent inflammation. Minor diverticulosis of the sigmoid colon was noted with no evidence of active diverticulitis. The patient subsequently underwent a laparoscopic appendicectomy during which an inflamed appendix as well as an inflamed epiploic appendage was found. Histology revealed a T-shaped appendiceal tip with the presence of two diverticula, showing features of acute appendicitis (Figures 9, 10). There was no evidence of malignancy.

Case 4

**Figure 11:**
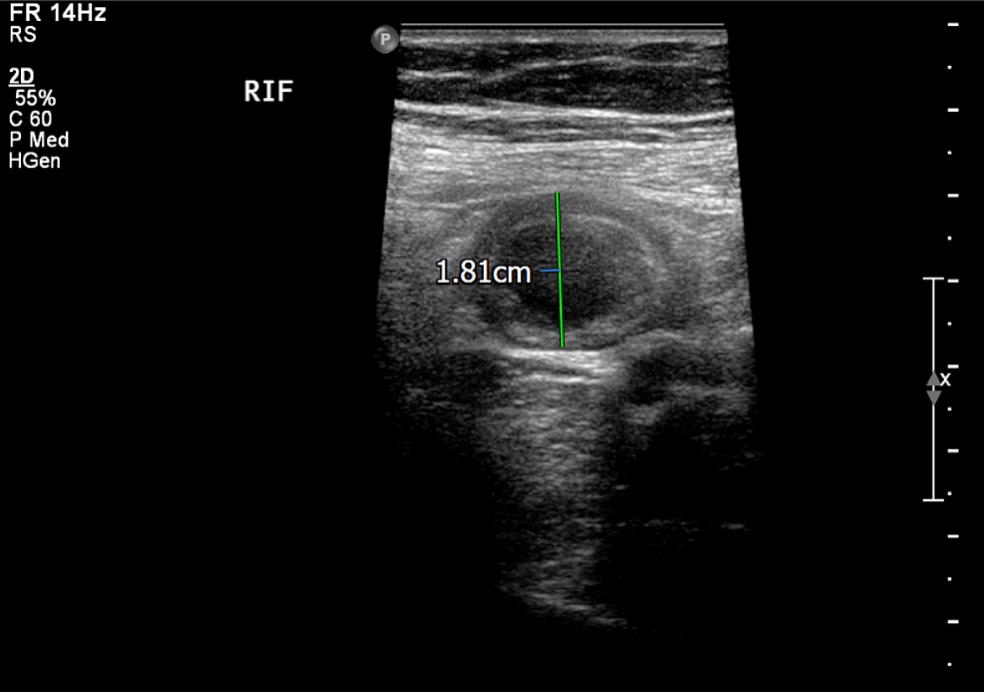
Abdominal ultrasound scan showing a thick-walled bowel loop (measuring up to 8 mm) suggesting inflammation.

**Figure 12:**
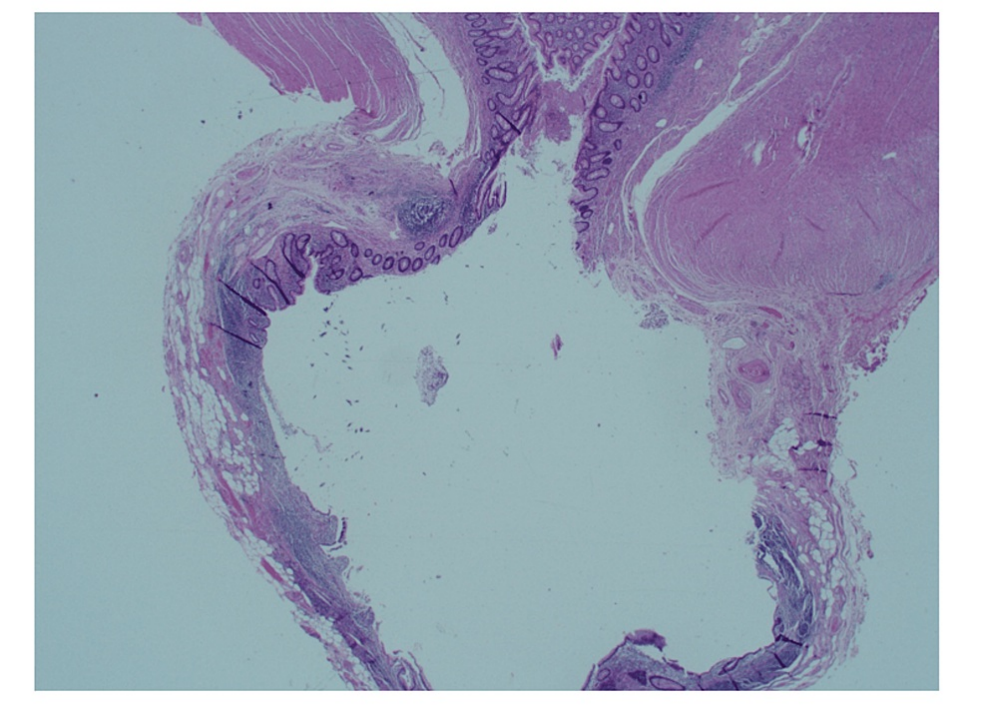
Large diverticulum sited deeply into an extremely thinned wall comprised mostly of fibrotic fibrofatty wall (x1.25). Hematoxylin & Eosin x1.25

**Figure 13:**
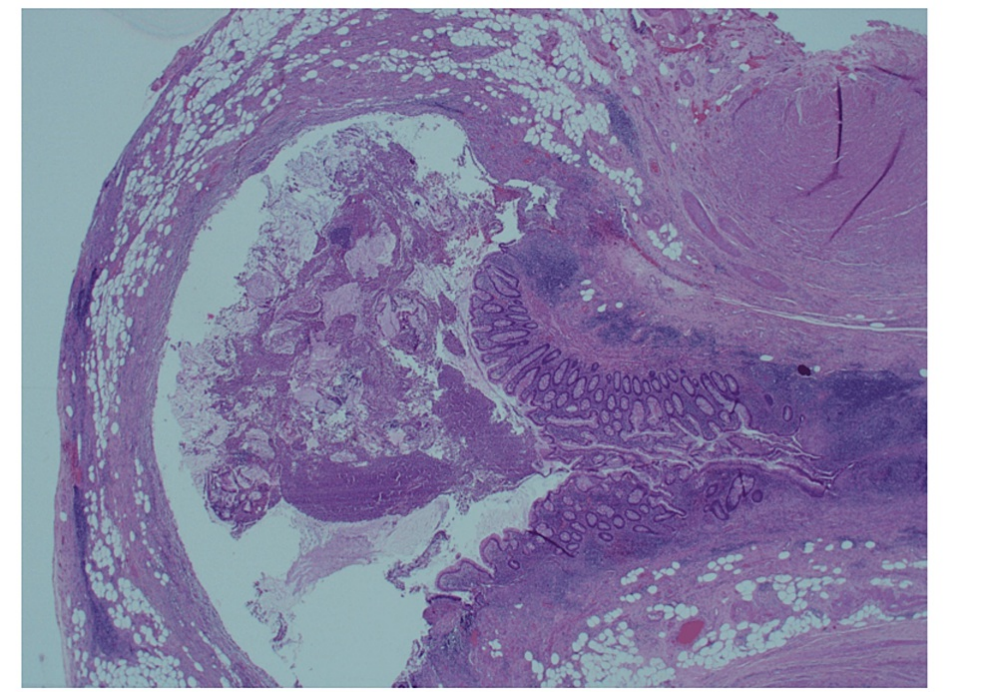
Large diverticulum sited deeply into an extremely thinned wall comprised mostly of fibrotic fibrofatty wall. Hematoxylin & Eosin x2.5

**Figure 14:**
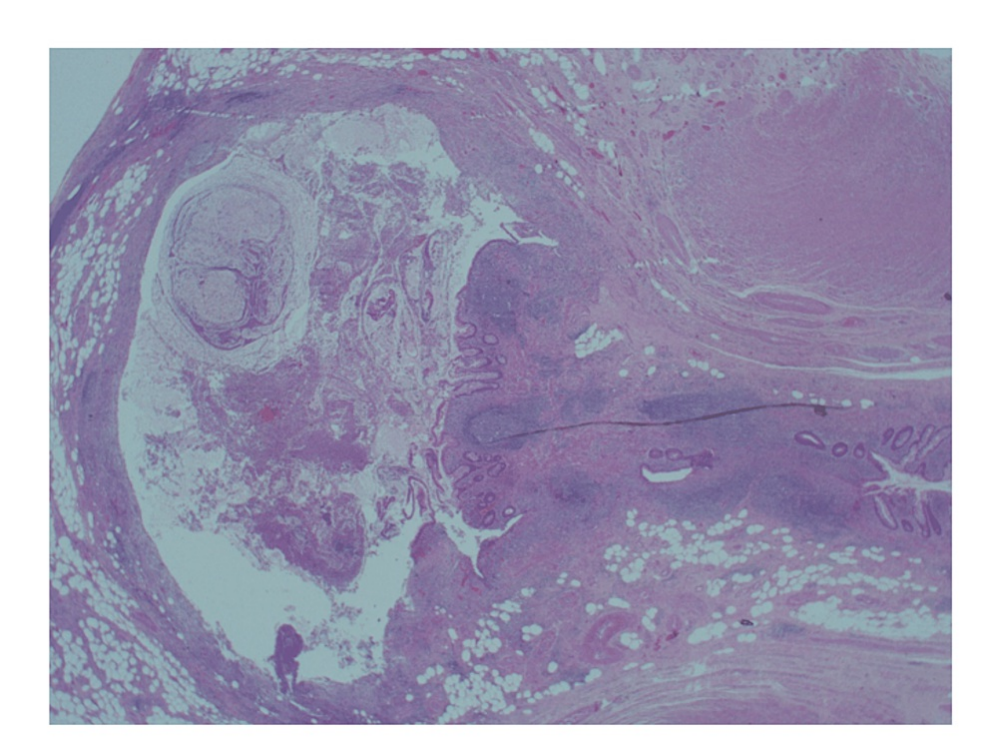
There is an intraluminal abscess (x4).

Patient 4 was a 47-year-old female who presented with RIF pain, anorexia, and diarrhoea. RIF tenderness was elicited on examination. Initial blood investigations showed a CRP of 8 mg/L, and a WBC of 5.8x10^9^/L. Ultrasonography of the RIF found a thick-walled bowel loop (measuring up to 8 mm) suggesting inflammation (Figure 11). It was difficult to conclude if this was the appendix or caecum. This patient subsequently underwent a laparoscopic appendicectomy which intra-operatively found an inflamed, retrocaecal appendix. The histology of this appendix showed an appendiceal diverticulum with acute inflammation (Figures 12, 13, 14).

Case 5

**Figure 15:**
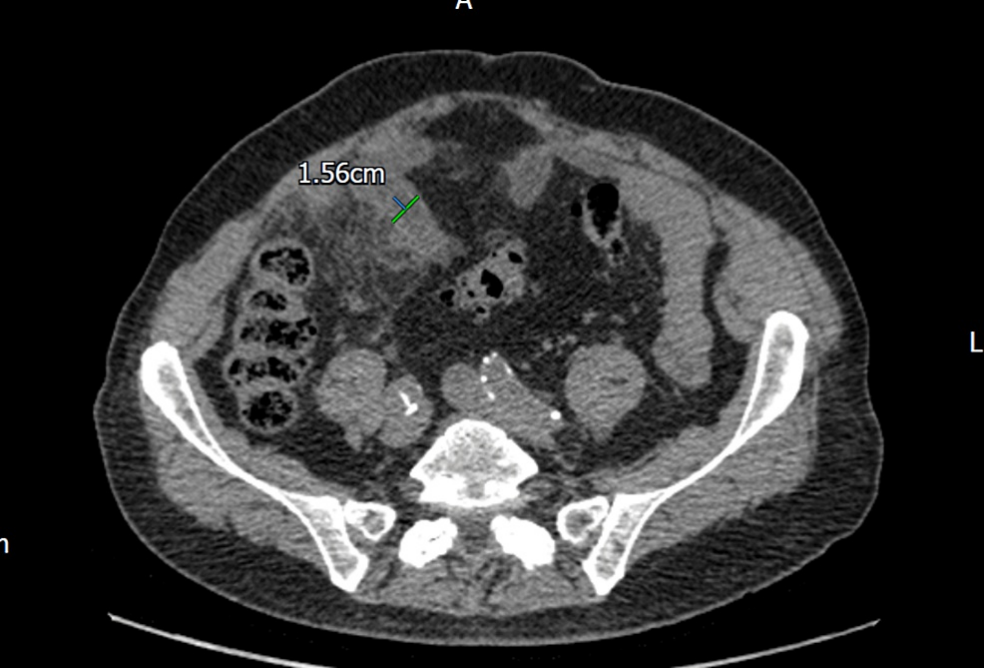
Axial CT scan - a highly suspicious ruptured appendix with acute appendicitis and focal changes.

**Figure 16:**
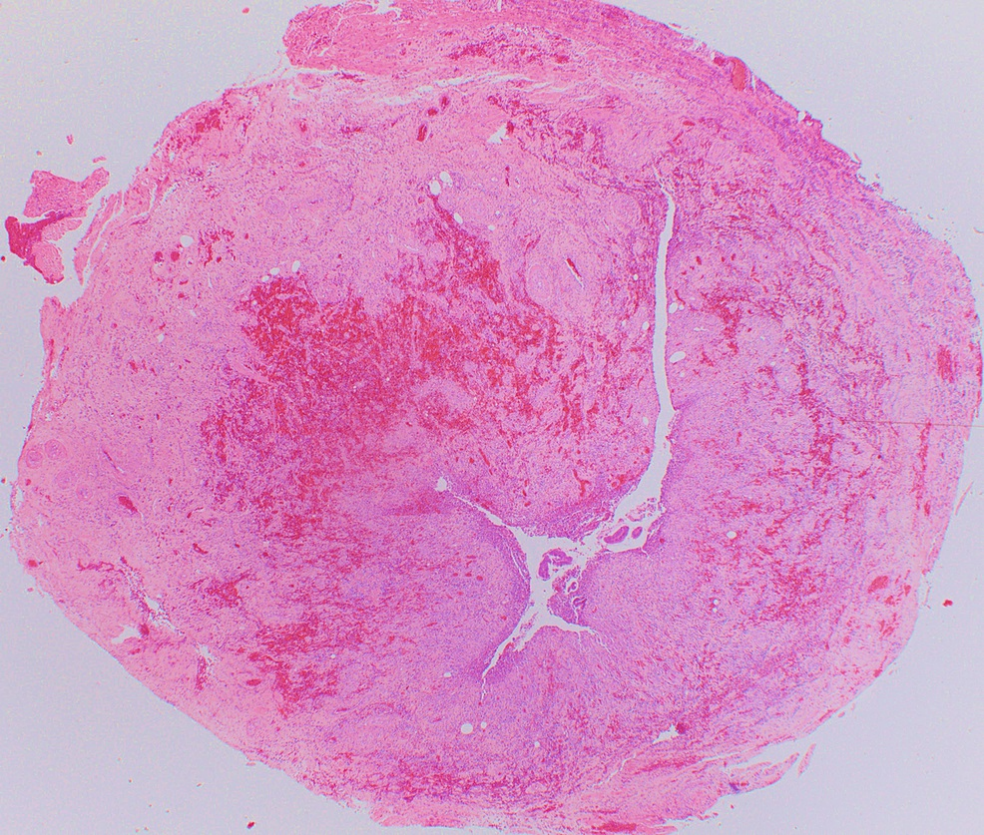
Transverse section of the appendix showing a linear extension of the diverticulum into the wall. There is extensive haemorrhage, necrosis, and acute inflammation. Hematoxylin & Eosin x20

**Figure 17:**
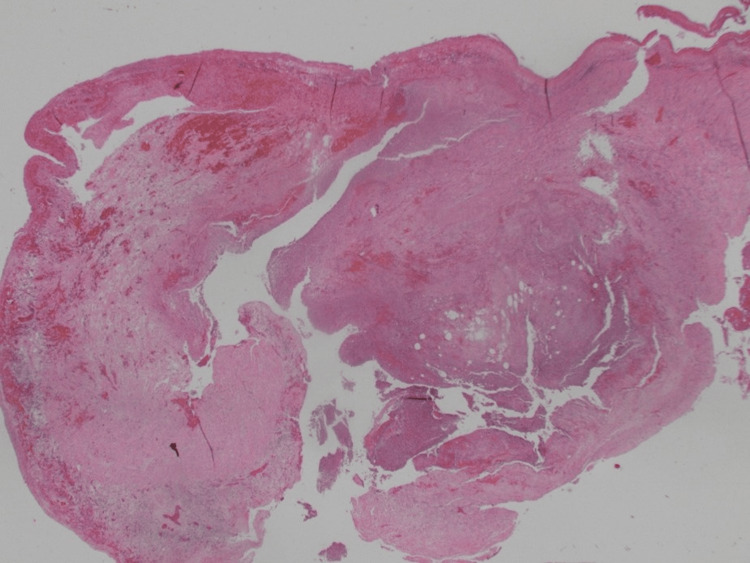
Longitudinal section of appendix towards the tip showing ruptured diverticular with tissue necrosis and acute inflammation. Hematoxylin & Eosin x12.5 (1.25 objective)

**Figure 18:**
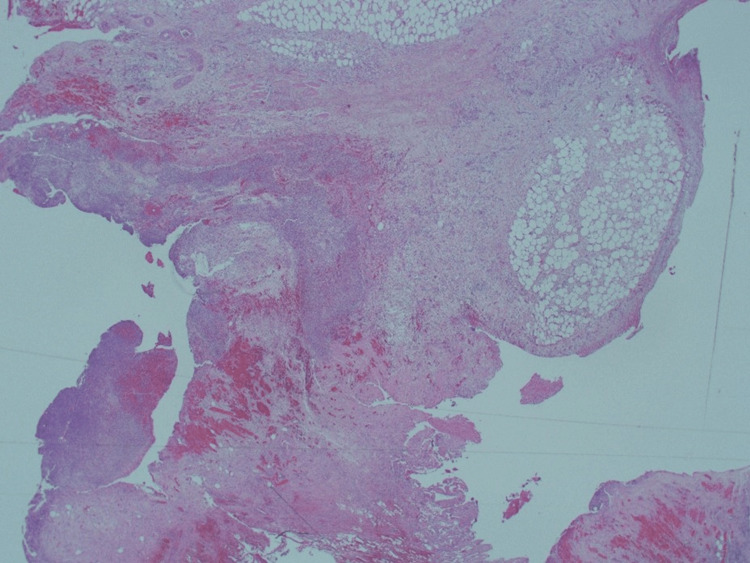
Figure shows the severity of acute inflammation, including intramural abscess. Hematoxylin & Eosin x40 (x4 objective)

Patient 5, an 84-year-old man, presented with a one-day history of lower abdominal pain and nausea. Initial blood investigations showed a CRP of 60mg/L and a WBC of 14.2x10^9^/L. CT scan showed a highly suspicious ruptured appendix with acute appendicitis and focal changes (Figure 15). An uncomplicated diverticular disease of the sigmoid colon was also noted. The patient subsequently underwent a laparoscopic appendicectomy. Intra-operative findings revealed a perforated appendix lying towards the pelvis with pus in the RIF and pelvis, associated dense adhesions between the appendix and mesoappendix, and loops of the small bowel and sigmoid colon. The histology showed features of acute gangrenous appendicitis, peri-appendicitis, and serositis. There was also a diverticulum present towards the tip of the appendix (Figures 16, 17, 18). He was investigated for anaemia in 2019 and the investigations revealed caecal cancer. He had a right hemicolectomy in 2020, which was fifteen months after his laparoscopic appendicectomy.

Case 6

**Figure 19:**
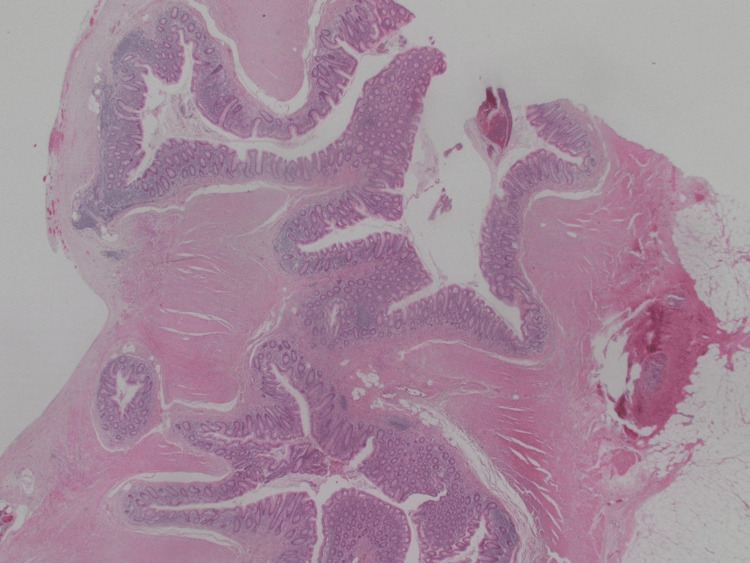
Longitudinal section toward the base of the appendix showing multiple diverticular protrusions through the wall. There is associated mild diverticulitis. Hematoxylin & Eosin x12.5 (objective 1.25)

**Figure 20:**
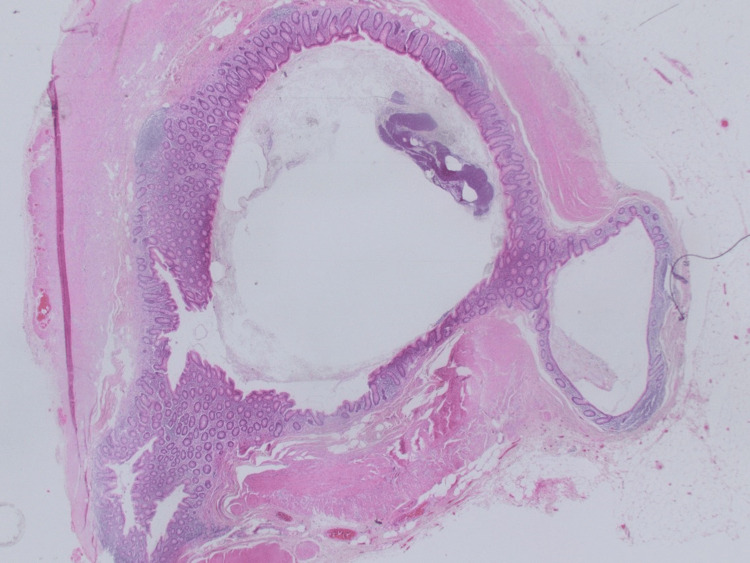
Transverse section of the mid-segment of the appendix also showing diverticular disease of the appendix. Hematoxylin & Eosin x12.5 (objective 1.25)

A 62-year-old man had an elective reversal of Hartman’s procedure in 2020. Intra-operatively, there was a presence of features suggestive of appendiceal diverticulosis, and an appendicectomy was done. Histology revealed a vermiform appendix with numerous out partings of the mucosa in keeping with appendiceal diverticulosis (Figures 19, 20).

The group included four males and two females, with ages ranging between 20 and 84 years. The most frequent presenting features were right iliac fossa pain, nausea, anorexia, and diarrhoea, except in the sixth patient, which was an incidental intra-operative finding.

The white blood count was raised in two cases (>11.0 × 10^9^/L), though none of the patients had leucopenia. The CRP was raised in all five symptomatic cases, however, significantly raised only in one case (288 mg/dl), moderately raised in three cases (>10-100mg/dl), and slightly raised in one case (<10 mg/dl). Three cases had pre-operative CT scans that revealed a thickened appendix with peri-appendiceal inflammatory changes in two cases (Figures 1, 2) and features of perforation in one case. The CT scan also detected uncomplicated diverticular disease of the sigmoid colon in the oldest patient.

The ultrasound scan was helpful in two cases, where it showed thickened appendix; this was suggestive of inflammation. All five symptomatic patients underwent laparoscopic appendicectomy, with the sixth patient having an open appendicectomy during an open reversal of Hartman's procedure. The intraoperative findings confirmed an inflamed/perforated appendix in five cases and a normal appendix in the sixth. The microscopic analysis also confirmed the features of acute appendicitis in all symptomatic cases in addition to a single inflamed appendicular diverticulum in three cases, two inflamed diverticula with T-shaped appendicular tip appendix in one case, multiple inflamed appendicular diverticula in one case (patient has very high CRP), and the presence of diverticulosis alone in the sixth. There was no evidence of parasites, dysplasia, or malignancy in the examined specimens (Table 1). 

**Table 1:** Clinico-pathological criteria of the six cases of diverticular disease of the appendix. RIF: right iliac fossa; CRP: C-reactive protein

No	Age (years)	Gender	History	WBC (x10^9^/L)	CRP (mg/L)	Imaging	Histology
01	40	M	RIF pain	14	288	CT: thickened appendix (13mm) with extensive perifocal fat stranding.	Transmural acute inflammation and serositis with multiple diverticula.
02	20	F	RIF pain	10.4	14	USS:thickened (8mm) and oedematous appendix.	Mucosal inflammation of the appendix not involving the muscle layer and an inflamed diverticulum.
03	70	M	RIF pain	10.7	19	CT:thickened appendix (10mm) associated with peri-appendiceal inflammatory changes. Sigmoid diverticulosis.	T-shaped appendiceal tip with the presence of two diverticula, showing features of acute appendicitis
04	47	F	RIF pain	5.8	8	USS:a thick-walled bowel loop (8mm) suggesting inflammation. It was difficult to conclude clear anatomy.	An appendiceal diverticulum with acute inflammation.
05	84	M	Lower abdominal pain	14.2	60	CT: highly suspicious ruptured appendix with acute appendicitis and focal changes.	of acute gangrenous appendicitis, periappendicitis and serositis. There was also a diverticulum present towards the tip of the appendix
06	62	M	Incidental	8.6	-	-	Numerous out partings of the mucosa in keeping with appendiceal diverticulosis.

## Discussion

There are two different types of appendiceal diverticula: congenital (incidence - 0.014%) and acquired (incidence - 1.9%). Acquired-type diverticula occur secondary to increased pressure within the appendiceal lumen, leading to an outpouching of the mucosa through the muscularis propria at low-pressure points along the mesenteric and antimesenteric borders [3]. Causes for this may include the presence of a fecalith, proximal tumours, and excess mucus [4]. The congenital type occurs due to the outpouching of all three appendiceal layers through a normal wall. This usually occurs on the antimesenteric border of the appendix and may be associated with diseases such as Patau syndrome [5].

Luc Deschenes et al. in 1971 described five morphological types [6]. The first is the presence of primary diverticulitis with or without peri-diverticulitis. Second is acute diverticulitis secondary to acute appendicitis. The third type is a non-inflamed, simple diverticulum discovered after a histological examination of the appendix. The fourth type is a simple diverticulum with acute appendicitis (the diverticulum is uninvolved in the inflammatory process). Lastly, the fifth type is the presence of chronic peri-diverticulitis with acute appendicitis [6]. The morphological classification of diverticular disease of the appendix was similarly described by Lipton et al. in 1989 [7]. They described four types: type I includes the presence of acute diverticulitis with a normal appendix, type II describes acute diverticulitis with acute appendicitis, type III is a non-inflamed diverticulum with appendicitis, and type IV is a non-inflamed diverticulum with a normal appendix. Types I-III are also sub-grouped into those with or without perforation [7]. Type I is the most frequently occurring, with a prevalence of 40-50% [8].

Risk factors associated with the development of this pathology include age more than 30 years and a history of cystic fibrosis or Hirschsprung’s disease [9]. The disease may present acutely due to the inflammatory process, similar to that of acute appendicitis. In other cases, it may present as a recurring, chronic pain or it may be completely asymptomatic [10]. The four cases described were seen in a range of age groups without any significant co-morbidity.

Yamana et al. in 2012 reported a lower white blood cell count and a high C-reactive protein level in patients with appendiceal diverticulitis compared to those with acute appendicitis [11]. This suggests that those with diverticulitis had a longer duration of inflammation at presentation than those with acute appendicitis [11].

Detecting the presence of appendiceal diverticulitis preoperatively is important in arriving at an accurate diagnosis. However, in most cases, the diagnosis is typically made during the histological examination of the appendix. Features of acute appendiceal diverticulitis on CT have been described in just a few publications. The presence of an inflamed diverticulum on CT is typically seen as a round, small cystic out-pouching with wall enhancement due to increased contrast uptake within the diverticular wall [12]. Lee et al. in 2007 found the presence of round, cystic outpouchings at the distal appendix, with contrast wall enhancement, in 80% of patients with inflamed diverticula. A finding of an appendicolith is rarely seen in contrast to patients with acute appendicitis [13]. Osada et al. in 2012 described CT findings of inflamed diverticuli, showing either small fluid-filled luminal structures with thick, enhanced walls or solid, enhanced masses protruding from the appendix, for six out of seven patients (confirmed to have appendiceal diverticula on histology) [14]. There is no documented association between colonic diverticulosis and appendiceal diverticulosis [15]. One of our patients did have an incidental finding of sigmoid diverticulosis on CT (Table 1).

Sonographic features of acute appendiceal diverticulitis include the presence of hypoechoic inflamed diverticula surrounded by echogenic fatty tissue. In typical, acute appendicitis, mucosal and submucosal inflammation can be visualised as an echogenic ring with an internal echo-free space filled with fluid. This differs from appendiceal diverticulitis wherein all the appendiceal wall layers are inflamed and visualised as thickened and echogenic with an internal area of echogenicity, which implies the presence of air [16].

Acute appendiceal diverticulitis may progress to several complications, which include perforation, intra-abdominal abscess, pelvic pseudocyst, and vesicocaecal fistula. Appendiceal diverticulosis may also lead to intra-abdominal haemorrhage and intussusception [2]. There is a high risk of associated appendiceal neoplasms such as carcinoid tumours and mucinous adenomas [17]. Cases have been reported of associated low-grade mucinous neoplasia, sessile serrated adenoma/polyp, carcinoid tumours and colorectal carcinoma, the last considered as the third most common malignancy after breast and lung and occupies the second place in the cancer-related mortality list (09.4%). Those lesions also are considered to be a differential diagnosis of appendiceal diverticulitis/diverticulosis [4, 18, 19-20].

Appendicectomy is the procedure of choice for acute appendiceal diverticulitis, and prophylactic elective appendicectomy is the recommendation for patients with diverticulum. This is due to the risk of progression to diverticulitis, perforation or malignancy. Clinicians should always remain cognisant of the differentials of acute appendicitis and keep appendiceal diverticulitis in mind when treating patients with right-sided, lower quadrant abdominal pain.

In this series, the clinical features of acute appendiceal diverticulitis were similar to those of acute appendicitis, and this was not diagnosed pre-operatively in those that had CT imaging. In looking at the literature, we find that it is possible to differentiate the two entities on CT and increased awareness is required on the part of radiologists. Despite the risk of neoplasm with this pathology, none of our patients had any long-term complications.

## Conclusions

Appendiceal diverticulitis is an uncommon pathology that imitates acute appendicitis and is usually treated by appendicectomy. Prophylactic appendicectomy should also be performed for non-inflamed diverticula. This is due to the risk of possible inflammation in the future, risk of perforation, and risk of developing an appendiceal neoplasm.
